# On the Rôle of Systemic Hyperacidity, and of Sulphocyanides in the Saliva, in Chemical Abrasion of the Teeth

**Published:** 1900-04

**Authors:** M. Michaels


### Abstracts and Translations.

### ON THE RÔLE OF SYSTEMIC HYPERACIDITY, AND OF SULPHOCYANIDES IN THE SALIVA, IN CHEMICAL ABRASION OF THE TEETH.[1]

BY M. MICHAELS.[2]

THE morbid origin and development of the chronic diseases grouped under the general name of the rheumatic diathesis many speculations have arisen to explain. While certain medical authorities deny the existence of the diathesis, others, in agreement with biological chemists, consider its recognition of peculiar value. According to these, it is distinguished both by a peculiar condition of impaired nutritive processes and by excessive acidity of the fluids of the body.[3]

The injurious matters which saturate the blood plasma, as the

---

[1] Paper read before the National Dental Congress of France, at the session at Nancy, August, 1898. Translated for The New York Institute of Stomatology by Drs. E. A. Bogue and C. O. Kimball, New York City.

[2] Honorary President of the National Dental Congress.

[3] Variations in acidity in the blood plasma have been studied indirectly through the urine by Gautrelet (Urines, Dépôts, Séd, etc., 1889); they have also, by Dr. R. Drouin, been directly demonstrated by analysis of the blood (Hemoalcalimetric, etc., 1893).

result of organic acidity, determine a special dyscrasia which affects all the organs and all the tissues, all secretions and excretions; hence the characters of this diathesis vary infinitely in their manifestations.

The phenomena of the chemistry of life expressed in the functions of physiological health are very complicated, and rest upon an equilibrium in biochemical reactions which is essentially unstable. The assimilation of food and the destruction of the body-cells, the chemistry of oxidation, of hydration, of combinations and resolutions,—all these at every moment are producing new changes and exchanges. If a disturbing influence enter any group of these chemical processes, disorder follows in others; of various kinds, but all betraying their presence in disease, according to the intensity of the process involved.

Gautrelet, in his book on urinary sediments and calculi and the application of urinary analysis to medical diagnosis, gives the following definition of diathesis:

"Careful study of the renal dialytic function demonstrates that the chemical reactions of the organism, to be normal, must take place in an environment,—that is, the blood serum, which contains salts acid in form and yet is alkaline in its reaction. On the other hand, the investigations of Duchaux have shown,—

"1. That alkalinity favors organic oxidation, while acidity retards it; and

"2. That all organic exchanges, in their last analysis, may be compared to fermentations, in which a variety of external substances, notably sodium chloride, will produce a diminution in their activity.

"Now, in chronic diseases, to keep watch of the changes in the blood plasma, noting both its variations in reaction and its variations from the normal percentage of its contained sodium chloride, this will be, we believe, to study the diathesis itself; for it will be an investigation of the essential factor in the morbid conditions,—that is, the departure from chemically normal blood.

"The urine, produced by cell-dialysis, reflects exactly the variations in the percentage contents of the blood, and to follow these step by step will be to obtain a really accurate conception of diatheses; which, according to the above suggestions, should be classified as,—

"1. Diathesis due to increased organic acidity; and

"2. Diathesis due to lessened organic acidity.[1]"

Expressed graphically, it is evident that a certain number of chronic diseases have this quality in common,—that there is an increase in the acidity of the urine.[2]

These maladies are grouped by Gautrelet under the general heading of the hyperacid diatheses;[3] and to demonstrate the causal relation of organic hyperacidity and its various manifestations, he describes the modifying influences of this biochemical acidity upon the derivatives of the blood plasma,—that is, upon the bodily secretions and excretions and the general chemical reactions of the organisms.

In the studies and observations which I have made of patients suffering from organic hyperacidity, I have invariably found the urine pathologically altered in a characteristic manner.

The elements which enter into the formation of urine as integral parts of it occur regularly in well-defined quantities and percentages, and any increase in the acidity of the fluid characterizes one form of diathesis; but the urinary elements in minute percentage characterize merely a tendency towards a particular diathesis. We may, therefore, draw conclusions as to the preparatory stages of a diathesis according as these urinary elements vary in their percentages, and according as we note the manifold abnormal evidences which

---

[1] Diminished acidity is a constant expression of physiological suffering. (Lallier.)

[2] The normal reaction of urine is more or less acid, and this acidity depends upon the contained acid phosphate of sodium. But the free organic acids should not be overlooked, especially lactic acid (derived from the muscles), which is at times present in considerable quantity. Other organic acids found in the urine are oxalic, hippuric, and phospho-glycerin. Carbonic acid adds to the general effect, though in less degree. This is Bouchard's conclusion from his study of the acid dyscrasias. How the urine can be acid when derived from an alkaline blood plasma we explain by supposing that the animal membranes, in dialyzing saline substances, allow more acid than basic salts to pass through. This has been verified for acid sodium phosphate. But why do not the other secretions of the economy become acid? Why can we not say that the acidity of urine appears only in the kidney because only there do its elements meet and unite, and that under the influence of their mixing there are formed, in the exchange of acids and bases, acid salts which do not pre-exist in this form in the blood? (Vieillard, L'Urine Humaine, p. 166.)

[3] Bouchard's valuable studies of the acid dyscrasias are of great assistance in the precise diagnosis of affections of the mouth and teeth in various conditions and diatheses.

may have but an indefinite relation to either the general or the special bodily condition.

The morbid chemistry of the arthritic diathesis resulting from the depressed vitality and the defective catabolism of the condition, saturates the blood plasma with acids of the fatty series, with acid urates,[1] and with their derivatives, producing thus certain diseased conditions by the changes effected in the bodily tissues and fluids, secretory and excretory. The result is an entire series of abnormal constitutional conditions, both primary and secondary, associated with gout, rheumatism, diabetes,[2] and other expressions of the arthritic diathesis.

As part of the accompanying phenomena in the morbid conditions mentioned we at times observe secondary effects, none the less characteristic, in the dental apparatus and the mucous membrane of the mouth. Among these are rheumatic inflammation of the gums, alveolar neuralgia, acidity of the mouth, looseness of the teeth, nervous palatal constriction, alveolo-dental abscess, expulsive gingivitis, chemical abrasion of the teeth, dental tartar and deposits of sordes, and dental caries of constitutional origin.[3]

In my observation of cases of affections of the buccal mucous membrane and of the peculiarities of dental caries among patients with hyperacidity, in view of differences noted among them I have been led to study individual variations, both in primary and secondary stages, and to follow the chemical changes in the saliva, urine, and sweat.[4] In such analyses of saliva and sweat, where the cases present chemical abrasion of the teeth, the percentage of acidity is less than among those who present clear cases of hyperacidity, as in gout and rheumatism, and the same is true on polariscopic examination for uric acid. On the other hand, oxalic acid appears in the urine as crystalline calcium oxalate, and the sulphocyanides of potassium and ammonium are found in the saliva in excess. In

---

[1] Organic alkaloids, and the urates, basic, acid, and neutral, are the waste products of tissue function. (Gautier.)

[2] Gautrelet considers the appearance of sulpho-cyanides in excess as a warning of the approach of diabetes in the hyperacid diathesis.

[3] I have found some of these conditions as primary also, without evidence of diabetes, but accompanied by excess of acidity. Urinary analysis, in doubtful cases, will determine the differential diagnosis.

[4] Human saliva, like the urine, is subject to physiological oscillations in its reaction, in constant relation with the chemical processes of the organism. (Vieillard.)

such patients the constitutional effects do not appear upon the gums, but dental erosion is unmistakable.

For the moment I do not assert that the salivary glands secrete sulphocyanides in excess; these elements are constant in the saliva of patients who may show no trace of chemical abrasion of the teeth. But in the case of others such dental affection is pronounced. The surface of the teeth is dissolved by molecules, but into no apparent necrosis, and this condition is produced by the chemical principles excreted by the follicles scattered over the epithelial lining of the mouth, especially of the lips. The more concentrated such chemical substance the more evident is the erosion.

Chemical dental erosion is undoubtedly of constitutional origin, and among rheumatic patients is characterized by peculiarities which have excited the interest and the therapeutic efforts of numerous observers; such are the absence of bacterial caries, the situation of the erosion upon the labial aspect of the incisors, the special form of the eroded areas, and the slow but certain destruction of the teeth attacked.

At first these abrasions are seldom accompanied with pain, but at times the loss of tooth-substance is so extensive that the parts are very sensitive. There is then, without doubt, a compensatory effort on the part of the pulp to secrete a secondary dentine; I have found incisors in which the pulp-cavity has become occluded by this new-formed dentine. In certain cases the hyperæsthesia is so intense that the lightest touch occasions intolerable pain.

The surfaces eroded are generally smooth and polished, and as their edges are sharp and their contour is peculiar, they may be mistaken for the effects of chemical abrasion.

In the "American System of Dentistry," Dr. Black publishes an article on abrasion and erosion of the teeth which concludes as follows:

"Dental erosion is an affection characterized by loss of substance of the teeth without apparent cause, varying in its form, but which may go on to almost total destruction of the dental crown.

"Such loss of substance occurs on the labial surface of the front teeth, and while certain forms may be produced by chemical action, others appear to be due to mechanical influences.

"The cause of such dental erosion is one of the most obscure problems of dental pathology, and our present information does not enable us to explain it.

"It is, therefore, the duty of each writer to consider this question of causation, and to base his studies upon earnest observation and experiment, in such fashion that we may hope for the solution of the problem."

Dr. Etchepareborda, of Buenos Ayres, in a communication to the International Dental Congress of 1889 (on the Influence of Rheumatism in the Production of Diseases of the Mouth and especially of the Teeth), handles the question from the view-point of the secondary effects of a diathesis, and his article deserves our attention. I quote certain affections of the mouth and the teeth. He has collected ninety cases in which there occurred the following buccal and dental diseases: Alveolar absorption alone, twenty-five cases; alveolar absorption with gingivitis, forty cases; alveolar absorption with osteo-periostitis, fifteen cases; spontaneous loss of teeth, eight cases; caries with alveolo-dental periostitis, two cases.

Etchepareborda concludes as follows:

"1. The teeth, the jaws, and the soft parts of the mouth are frequently the seat of maladies of rheumatic origin.

"2. These may accompany, precede, or follow the rheumatic manifestations in joint, muscle, and fibrous tissue, acute, subacute, or chronic; or they may remain for a long time the only visible expression of the diathesis.

"3. The most frequent effects of rheumatism are,—(*a*) of the teeth: alveolar and alveolo-dental periostitis, dental necrosis, and spontaneous loss of teeth; (*b*) of the gums: simple or aphthous inflammation; (*c*) of the nerves: facial neuralgia; (*d*) of the jaws: alveolar absorption, caries, and necrosis of the maxillary bones.

"4. Though any one of these local affections may be considered as rheumatic, they are also observed, in the same chemical form, among patients who are not rheumatic; in other words, their origin may be either local or constitutional. It is impossible to determine their place in the chronological order of rheumatic manifestations, but in general they appear late. Their maximum frequency occurs in middle life, that is, between twenty-five and forty years of age.

"5. It is especially in the indefinite and chronic forms that we observe these rheumatic affections. They often alternate with each other. In chronic cases, for example, a subacute attack involving the fibrous structures of the joints, or the muscles, is often followed by decrease in the affections in the mouth. These cases may be treated without fear of causing local trouble of the same kind or more serious; and, indeed, it is necessary to treat them on account of their steady progress. In considering the therapeutic indications, one must never forget the foundation of constitutional rheumatism upon which they rest, for they demand not only local attention, but general treatment as well."

My experience is corroborated by Dr. Rosenthal, of Nancy, in his paper on "Secondary Changes in the Dental Apparatus;" noting, as he does, the effect of constitutional conditions upon the teeth, and I am glad to acknowledge the value of his article; his cases are well chosen and characteristic of various pathological states of the teeth. Farther on in this monograph, and fortifying my position by the special chemical study of the saliva and the urine, I shall endeavor to probe still deeper into this matter; and I do not doubt that more complete knowledge of the saliva in diatheses would put us in possession of more radical methods for the cure of dental diseases.

The expression "chemical erosion" of the teeth, according to Bödecker ("Anatomy and Pathology of the Teeth"), "designates a process by which the hard tissues of the tooth are scraped and destroyed, chiefly on the labial surface near the neck of the tooth. It is marked by loss of substance in the tooth, occurring without apparent cause. It begins in a small spot on the surface of the tooth, a hollow or groove is formed which little by little increases till often a large area is destroyed, and this lesion is situated on the labial surface of the crown and most often on the front teeth.

"The authors who have studied erosion term it chemical abrasion and destructive denudation, which are synonymous. Such erosion consists in an excavation of the enamel of certain of the front teeth, usually situated midway between the neck and the biting edge of the tooth, but sometimes at other points on the crown.

"At first the incisors, then the bicuspids, are most frequently attacked, either of upper or lower jaw; and the erosion is not limited to one tooth, but progressively involves others, appearing in about the same place as on those first invaded. The disease advances with time, and the amount of destruction depends upon the epoch of the primary attack. The form of the abrasion does not vary on one tooth or the other, for the second and third are injured as was the first; at the same time, it is seldom found that the trouble is of equal extent upon different teeth except in the case of the central incisors.

"The area destroyed presents many variations in its relation to other and normal portions of the tooth. Usually the form of the erosion is a vertical triangle, the apex towards the pulp and the base peripheral. But while towards the free border of the tooth there is an angle of varying sharpness, and an incline leads rapidly to the bottom of the erosion, towards the gum the area tapers gently upward and ends as a shallow groove near the neck of the tooth. The eroded surface is smooth and glistening, or, in some cases, rough and opaque, and this latter appearance is due to a beginning caries. The color of the part at first is white or yellowish white, from reflection from the dentine and cement; but at last it becomes dark yellow or even black.

"The pathological anatomy of the lesion requires no special description; under the microscope the same appearance is presented as in the case of mechanical abrasion. There is a formation of secondary dentine in the pulp-cavity which explains the great sensitiveness of such teeth."

This is the description of chemical erosion or abrasion which is given by the American author, which I desire to complete by calling attention to certain characteristics of this malady and especially its relation to the labial glands.

Certain specimens in my possession present the following points of interest:

These chemical abrasions vary infinitely in their outline and depth, but their situation depends always upon the position of the labial glandules, which, as we shall see, are the secreting organs—in some pathological conditions to be more precisely described below—of the active chemical agent which produces the erosion. One type of this affection, according to my observation, may be described as an intermittent contact of the glandular excretion with the surface of the tooth, by which the enamel is dissolved by degrees. The lesion does not extend far at first, but merely hollows the tooth, owing to the relation of the excretory orifices of the gland. At this time, according to my specimens, the eroded surface has terraced borders and smooth bottom, and the natural color is retained. In time the erosion increases superficially and in depth, and even undermines the enamel; for the active principle flows in various directions and thus produces the defect. Usually the edges are sharply cut, but they may be dull and rounded. The appearance of caries is so unlike the above that it is not possible to confound the two conditions.

The number of the teeth involved may be small, limited to one or two, or all the front teeth are attacked. In a case reported (Fig. 2), the right canine is scooped out on its entire labial aspect to nearly the whole thickness of the tooth, and resembles in form the mouth of a flute.

The destructive process lasts a long time, for the active agent of the process is excreted only in infinitely small amounts, and the contact between tooth and gland is intermittent; and the feebleness of the chemical action is an additional cause.

(To be continued.)

---

